# Interdependent Multi-Layer Networks: Modeling and Survivability Analysis with Applications to Space-Based Networks

**DOI:** 10.1371/journal.pone.0060402

**Published:** 2013-04-11

**Authors:** Jean-Francois Castet, Joseph H. Saleh

**Affiliations:** Georgia Institute of Technology, Atlanta, Georgia, United States of America; Universidad de Zarazoga, Spain

## Abstract

This article develops a novel approach and algorithmic tools for the modeling and survivability analysis of networks with heterogeneous nodes, and examines their application to space-based networks. Space-based networks (SBNs) allow the sharing of spacecraft on-orbit resources, such as data storage, processing, and downlink. Each spacecraft in the network can have different subsystem composition and functionality, thus resulting in node heterogeneity. Most traditional survivability analyses of networks assume node homogeneity and as a result, are not suited for the analysis of SBNs. This work proposes that heterogeneous networks can be modeled as interdependent multi-layer networks, which enables their survivability analysis. The multi-layer aspect captures the breakdown of the network according to common functionalities across the different nodes, and it allows the emergence of homogeneous sub-networks, while the interdependency aspect constrains the network to capture the physical characteristics of each node. Definitions of primitives of failure propagation are devised. Formal characterization of interdependent multi-layer networks, as well as algorithmic tools for the analysis of failure propagation across the network are developed and illustrated with space applications. The SBN applications considered consist of several networked spacecraft that can tap into each other's Command and Data Handling subsystem, in case of failure of its own, including the Telemetry, Tracking and Command, the Control Processor, and the Data Handling sub-subsystems. Various design insights are derived and discussed, and the capability to perform trade-space analysis with the proposed approach for various network characteristics is indicated. The select results here shown quantify the incremental survivability gains (with respect to a particular class of threats) of the SBN over the traditional monolith spacecraft. Failure of the connectivity between nodes is also examined, and the results highlight the importance of the reliability of the wireless links between spacecraft (nodes) to enable any survivability improvements for space-based networks.

## Introduction

In many engineering disciplines, analyzing and modeling potential failures is a central focus for system design and operations. Given the development of increasingly complex and interconnected systems, it has become even more important to assess their propensity to failures and examine how local node failures would propagate throughout a networked system. For example, will the network experience catastrophic cascading failure? Will it exhibit graceful degradation? Or will the local failure remain confined to the node's neighborhood and not affect the system level performance? What design features are associated with each of these failure behaviors? These concerns fall within the realm of survivability analysis. Survivability is extensively used in the technical literature as a multi-disciplinary concept in a variety of contexts [1]. It is context-specific, related to the system studied and its environment, the services it provides to users, and the requirements that have been set for it. Roughly speaking, survivability of an engineering system is related to its performance degradation following a shock or disruption, the more survivable a system (with respect to a specific threat), the smaller the drop in the performance metric of interest. Similarly, recoverability and resiliency add a temporal dimension to the definition of survivability by accounting for the time it takes to return to the pre-shock level of performance. Only survivability is considered in this work and details will follow in the next sections.

This work is at the intersection of three strands of thoughts and research areas: network (survivability) analysis; interdependent networks (modeling and analysis); and space-based networks. We briefly examine next each of these areas to provide a background and motivation for our study. The present work makes a contribution to the modeling and survivability analysis of networks with heterogeneous nodes, which we propose can be mapped into multi-layer interdependent networks. We develop formal characterizations of interdependent multi-layer networks and algorithmic tools for the analysis of failure propagation across such systems. We then apply our approach to the case of space-based networks and we assess the survivability increments or gains of the network architecture over the traditional monolithic spacecraft design.

Networks are widely studied in the scholarly literature, as they describe a large number of technical, biological, and social systems: the World Wide Web and the Internet, power grids, telecommunications systems, social relationships, food webs, to cite a few [2], [3]. Networks have also been studied with the specific focus on failure propagation and cascading failures [4], [5], [6], [7], [8], [9]. For example, Crucitti *et al*. [5] described a model for cascading failures in communication/transportation network by dynamically redistributing the flow on the network after the failure of a node, this redistribution leading to the overload of other nodes in a cascading fashion. More recent analyses noted that the failure behavior of many modern networks cannot be independently studied as these networks are coupled together. For example, the electrical power grid and the Internet rely on each other for communication and control on one hand, and electricity supply on the other hand [8]. A failure in one network can have repercussions in the other, and these analyses showed that while an independent single network will break down after the removal of a significant number of nodes, interdependent networks can fail catastrophically after the removal of a small fraction. This approach led to the introduction of interdependent network analyses to characterize properties of such networks [8], [10], [11], [12], [13], [14], [15], [16]. For example, Kurant and Thiran [12] introduced the concept of a two-layered network to study the dynamics of a transportation system. The authors noted that the representation of such systems as a single network is inappropriate, as it does not allow the modeling of both the physical topology of the network and the traffic flow on it. In a similar vein, Xu *et al*. [16] developed the concept of interconnecting bilayer networks, where networks on two layers can share some common nodes (e.g., the networks of scientists and musicians can share similar persons, as a person can both be a scientist and a musician). In short, there is a growing interest and contributions to interdependent network analysis, and a budding attempt to account for and model both the physical characteristics of the networks and the functions they perform (their abstraction).

One common feature across many of these studies is the assumption of homogeneous nodes in the network, that is, all nodes perform identical functions and can thus potentially substitute for each other. This assumption, when justified, simplifies the analysis and enables the handling of a significantly large number of nodes. But in some cases as we will see shortly, this assumption is not justified and the previous approaches are not applicable to the analysis of networks with heterogeneous nodes, that is, nodes performing different functions. Some attempts at considering heterogeneous nodes have been proposed in the literature. For example, a few studies considered nodes with different capacities [4], [5], but the function of the nodes remains identical. The Internet network for example raises questions about heterogeneity as it consists of different networks (wireless devices, computers, routers, etc.). However, the emphasis in studies of the Internet is primarily on the transmission of data among the nodes rather than on the heterogeneity in networks. In short, modeling node homogeneity is prevalent in network analysis, and while some recent studies do account for node heterogeneity, the “extent” of heterogeneity modeled remains confined to variations on the same function performed by all the nodes. These approaches are not applicable for our application of interest, namely space-based network in which nodes (spacecraft) can be significantly distinct from each other, and thus considerably affect failure propagation throughout the network. We briefly describe next what space-based networks (SBNs) are.

**Figure 1:**
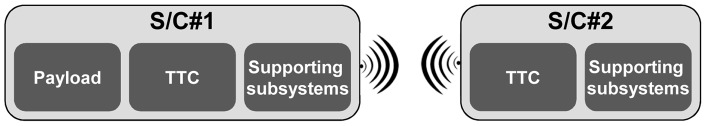
Example of a space-based network.

SBNs are related to a novel concept recently introduced in the space industry termed fractionation [17], [18]. By physically distributing functions in multiple orbiting modules wirelessly connected to each other, this new architecture allows the sharing of resources on-orbit, such as data processing, data storage, and downlinks. Preliminary analysis suggests that such an architecture, under certain conditions and despite some initial overhead, offers several advantages over the traditional monolith spacecraft design in terms of flexibility, responsiveness, and overall utility [19]. One of the initial motivations for the present work was to assess and benchmark the survivability of a fractionated architecture against that of a traditional monolith spacecraft. Spacecraft in an SBN can have different components due to the fractionation of the functionality, resulting in node heterogeneity. To illustrate this point, consider the simple example of a space-based network consisting of two networked spacecraft that can tap into the other's Telemetry, Tracking and Command (TTC) subsystem in case of damage or failure of its own TTC. This architecture is shown in Figure 1. The wireless connectivity in the SBN enables a new type of redundancy, functional but not co-located, of the TTC between the two spacecraft in the network. Each spacecraft is composed of the following subsystems (see Wertz and Larson [20] for details about these subsystems):

- The first spacecraft, S/C#1 contains all subsystems typically found in a spacecraft. For an easier representation, S/C#1 is composed of three “components”: a payload component (the instrument or set of instruments the spacecraft was designed to carry to fulfill its mission and generating utility), a TTC component (linking the spacecraft to the ground station and operators, enabling the proper tracking of the spacecraft, the monitoring of its subsystems and the upload of commands from the operators), and a “supporting subsystems component” composed of the remaining subsystems (Attitude and Orbit Control Subsystem (AOCS), Electrical Power Subsystem (EPS), Beam and Antenna, Control Processor (CP), Structures and Mechanisms) and which is necessary for the operation of the spacecraft.
- The second spacecraft, S/C#2, is composed of a TTC component and a supporting subsystems component (equivalent of the one of S/C#1). Note that S/C#2 has no payload component, and it is envisioned as a backup for S/C#1's TTC. This is just one example of an SBN and other configurations and spacecraft (node) components can be considered.

This example is provided for illustrative purposes to clarify the issue of node heterogeneity and how SBNs enable a non co-located redundancy of the TTC in this case. It may be objected to this example on practical grounds that the redundant TTC can simply be included in spacecraft #1 and yield the same reliability or survivability gains without the additional cost of spacecraft #2. For spacecraft, co-located redundancies maintain the same vulnerability of the system to a strike by orbital debris or anti-satellite weapons (ASAT). As such, an SBN, even the illustrative example provided, does offer some advantages in dealing with these types of threats over the traditional monolith spacecraft. These advantages in turn have to be carefully assessed and traded against the incremental costs of obtaining them.

**Figure 2:**
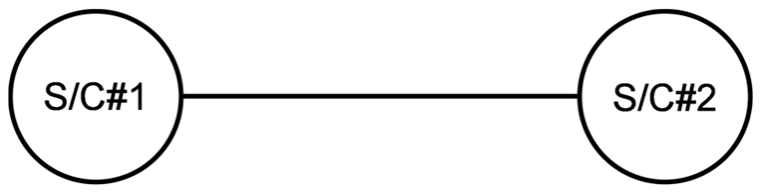
Inadequate representation of the SBN.

It is clear that if we were to represent this particular SBN as shown in Figure 2, the nodes cannot be considered identical and do not share the same functions, since S/C#1 possesses a payload component, while S/C#2 does not. In addition, the connectivity shown in Figure 2 would be ambiguous as some subsystems on-board the spacecraft share resources (TTC) while others do not (supporting subsystems). In short, the heterogeneity of the nodes in the SBN in Figure 1 cannot be captured and modeled by the traditional network analysis tools and representations.

In response to these issues, we propose to contribute a novel framework and analytical tools for the modeling of networks with heterogeneous nodes, and apply them to space-based networks with the objective of assessing their survivability. The remainder of this article is organized as follows. Section 2 develops the concept of interdependent multi-layer networks and provides a formal mathematical characterization. Section 3 examines the mechanisms of failure propagation across interdependent multi-layer networks. Section 4 provides survivability results for select case studies and demonstrates the capabilities here introduced by examining the survivability of space-based networks. Section 5 concludes this work.

## Interdependent Multi-Layer Networks: Formal Characterization

### 2.1. General Definition of Interdependent Multi-Layer Networks

**Figure 3:**
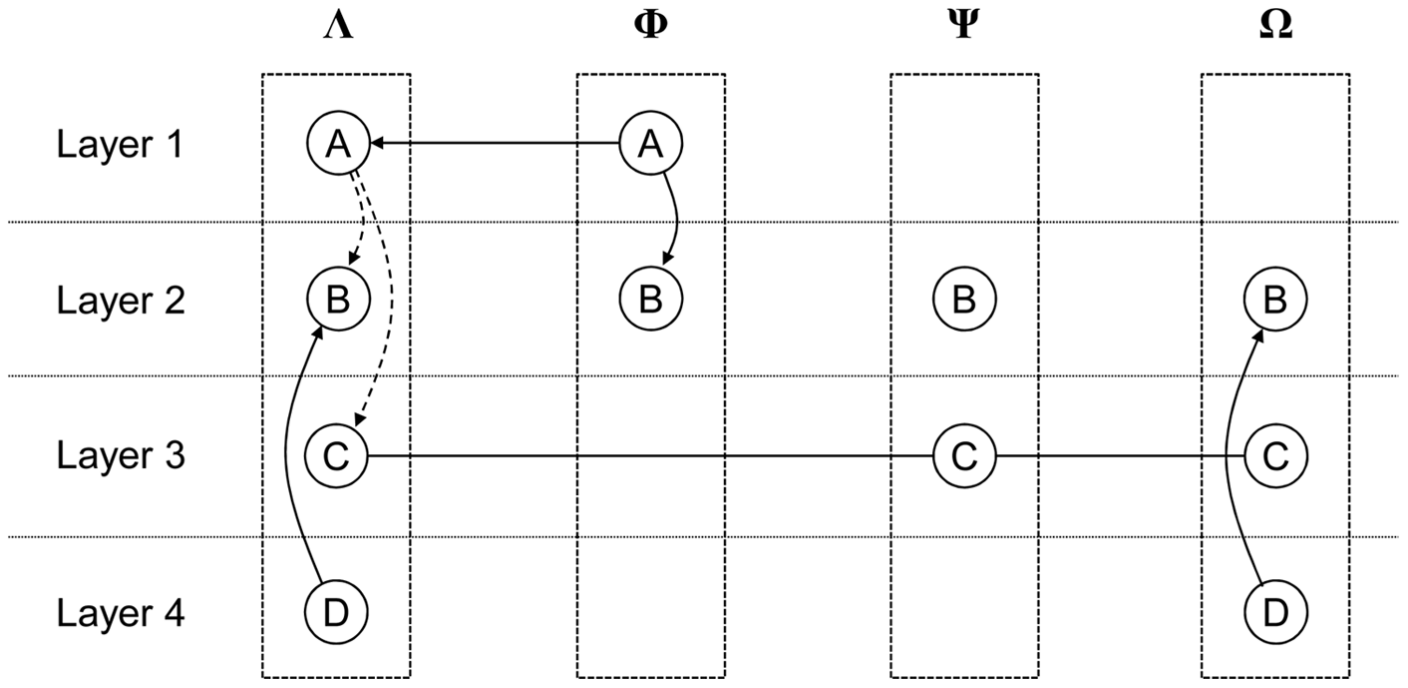
General representation of an Interdependent Multi-Layer Network.

Building on the concepts of interdependency and layers in network discussed previously, we propose to model a network with heterogeneous nodes as an interdependent multi-layer network (IMLN). We first introduce the following terminology, illustrated by the general network depicted in Figure 3 with four heterogeneous nodes labeled Λ, Φ, Ψ and Ω, with four possible functionalities labeled A, B, C and D.

- *Super-node*: heterogeneous node in a network that can have different functionalities. For example, Λ is a super-node. A spacecraft in a SBN for instance is a super-node;
- *Node*: component of a super-node that represents a single functionality. For example, the circle labeled A in Λ is a node. The TTC subsystem for instance is a node in a SBN;
- *Layer*: collection of nodes that have the same functionality. For example, Layer 1 in Figure 3 captures the functionality A across all the super-nodes in the network. For example, all the TTC subsystems in a SBN constitute a layer;
- *Intra-layer link*: link present between two nodes in the same layer if there is a connection between these nodes (e.g., flow of data, or in this work, a node that provides resources to another one). An intra-layer link can be directed (from the node that provides the resources to the receiver node) or undirected (which can be conceived as two opposite directed arcs, i.e., both nodes provide resources to the other). For example, Layer 1 in Figure 3 has a directed intra-layer link, indicating that super-node Φ provides functionality A to super-node Λ. Layer 3 has two undirected intra-layer links between nodes of functionality C (across the super-nodes Λ and Ψ, and Ψ and Ω). Note that in Layer 3, the super-node Λ does not provide the functionality C to super-node Ω, as there is no explicit intra-layer link between them (i.e., intra-layer links in this work do not have transitive properties);
- *Networked layer*: layer that possesses intra-layer links. For example, Layer 1 and 3 are networked layers, whereas Layer 4 is not;
- *Inter-layer link*: directed link that captures interdependencies between functionalities within a super-node. In this work, interdependency refers to the transmission of failure and two primitives of failure propagation are explored: the “kill effect” and the “precursor effect”:
  - The “kill effect” represents an immediate transmission of failure and is symbolized in Figure 3 by a solid arc. For example in the super-node Λ, the failure of the node representing the functionality D immediately results in the failure of the node representing the functionality B;
  - The “precursor effect” represents a delayed transmission of failure and is symbolized in Figure 3 by a dashed-line arc. For example in the super-node Λ, the failure of the node representing the functionality A may result in the failure of the nodes representing the functionality B and C, but not necessary immediately (conditional propagation of failure). As the node A in Λ is connected to a similar node in Φ, the super-node Λ will lose the functionality A only when both nodes have failed, or when the node in Λ has failed and the intra-layer link in Layer 1 between Λ and Φ has failed. In effect, the network for the functionality A allows the survival of the super-node Λ by tapping into the resources of another super-node (Φ). Note that because super-node Λ does not provide resources to super-node Φ, the failure of the functionality A in super-node Φ is immediately propagated to the node B through a kill effect scheme.
- Note that failures of the nodes representing the functionalities B and C have no impact on other functionalities. As a side note, different types of interdependencies between layers have been used in the literature: for example, Zio and Sansavini [9] used interdependency links to transfer loads from a failed node, or Gu et al. [21] used interdependent links to model cooperation between sub-networks. The interdependency scheme used by Buldyrev et al. [8] is similar to the kill effect described in this work. Note that the two effects presented in this work are not meant to be exhaustive, and other cascading failure mechanisms can be easily added and implemented.

These general definitions are applied to a specific space-based network in section 2.3 as an illustrative example.

In summary, the IMLN representation consists of nodes placed on several layers representing different types of functionalities. Within each layer, nodes form a network by connecting to other nodes with directed or undirected intra-layer links. In addition, inter-layer links connect nodes across layers to capture the physical reality of super-nodes and model various types of interdependencies. In this work, the interdependencies of interest (for survivability analysis) are two primitives of failure propagation, here termed the kill effect and precursor effect. A formal mathematical characterization of interdependent multi-layer networks is presented next.

### 2.2. Formal Definition of Interdependent Multi-Layer Networks

Building on the notation of Gu *et al*. [21], the interdependent multi-layer network *N* is defined as 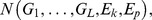 where:



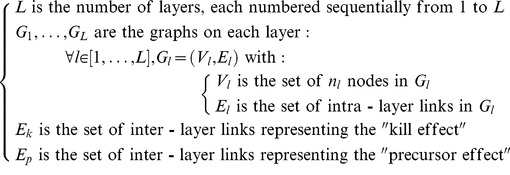
(1)


The set of networked layers *E_R_* is defined as:
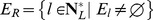
(2)


The total number of nodes in *N* is 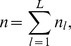 and the nodes are numbered uniquely and sequentially from 1 to *n*. As indicated in Newman [2], “it does not matter which vertex gets which label, only that each label is unique so that we can use the labels to refer to any vertex unambiguously.”

A more practical representation of the network *N* is given by using: 1) the classic adjacency matrices 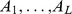 for the respective graphs 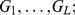 2) what is introduced in this work as the “inter-layer” matrix *C*; and 3) a mapping function *f* between two node numbering schemes described next.

The nodes are numbered from 1 to *n*. This numbering scheme is referred to in this work as the “*overall numbering*” and is used as the primary numbering scheme. An additional numbering of the nodes is introduced to define adjacency matrices, called the “*layer numbering*”: for each layer *l*, the nodes are numbered sequentially from 1 to *n_l_*. The function *f* maps the labels *k_O_* of each node in the “overall numbering” scheme to a pair of integers 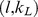 where *l* is the layer number, and *k_L_* is the label of the node in the “layer numbering”. Note that indices in the “overall numbering” scheme have a subscript “*O*”, while the indices in the “layer numbering” scheme have a subscript “*L*”. Because of the layers and the nodes in both numbering schemes are numbered uniquely, the function *f* is bijective. As a consequence, the inverse mapping function 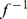 is also defined.

For each layer *l*, the graph *G_l_* can be represented by the associated adjacency matrix 
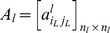
 such that:
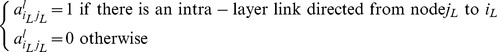
(3)


The “inter-layer” matrix 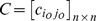 is defined as follows:
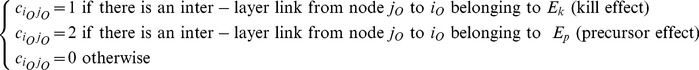
(4)


The sets *E_k_*, *E_p_* and *E_R_* can also be defined from the adjacency matrices and inter-layer matrix as follows:
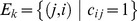
(5)

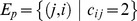
(6)

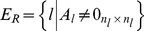
(7)


In summary, the interdependent multi-layer network *N* can be uniquely defined as 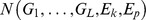 or 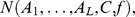 as the two characterizations are equivalent. We will use both hereafter, and will illustrate some advantages for the latter in the following section.

### 2.3. Illustration of Interdependent Multi-Layer Networks

**Figure 4:**
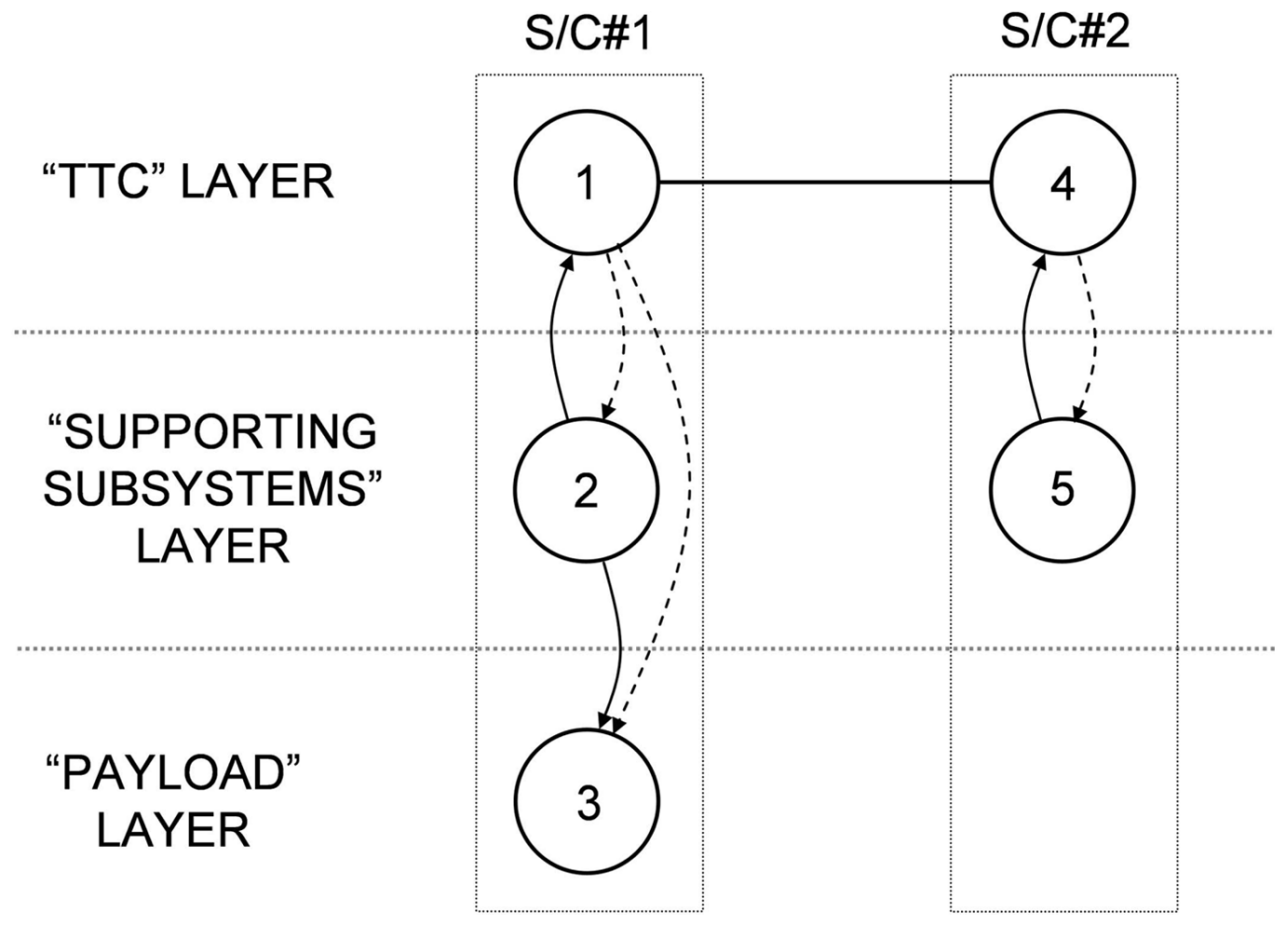
Interdependent multi-layer network representation for the example SBN.

The proposed representation is illustrated in this subsection using the space-based network presented in Figure 1 and its IMLN representation is given in Figure 4. Two spacecraft are part of the network and are represented by two super-nodes. The three identified functionalities in this particular SBN are the payload, the TTC and the supporting subsystems. Three layers are then created to represent each of these functionalities, as shown in Figure 4. The failure of the supporting subsystems results in the immediate failure of the whole spacecraft, leading to the unavailability of other nodes (TTC, payload) in different layers belonging to that spacecraft. Consequently, it falls under the “kill effect” type of interdependency within a super-node. The failure of the TTC does not necessarily result in the immediate failure of the spacecraft. The functional redundancy on the TTC can allow the survival of the spacecraft if it can tap into the TTC of the other spacecraft. This is possible if, in the TTC layer, both the link to another TTC node and that TTC node are functioning. Consequently, the failure of a TTC node falls under the “precursor effect” type of interdependency. As a result, we have:

- In the case of S/C#1, the “supporting systems” node failure renders unavailable both the “TTC” node and the “payload” node through the “kill effect”. The “TTC” node renders unavailable the “supporting subsystems” node and the “payload” node through the “precursor effect”. The “payload” node failure has no impact on the other nodes as the loss of the payload does not doom the spacecraft, only its ability to generate utility.
- In the case of S/C#2, failure of the “supporting systems” node renders unavailable the “TTC” node through the “kill effect”. The “TTC” node renders unavailable the “supporting subsystems” node through the “precursor effect”.

For this particular SBN, the interdependent multi-layer network is mathematically defined as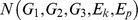 where:

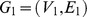
 with 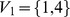 and 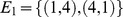 is the graph for the “TTC” layer (layer 1);

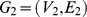
 with 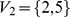 and 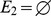 is the graph for the “supporting subsystems” layer (layer 2);

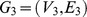
 with 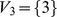 and 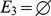 is the graph for the “payload” layer (layer 3);

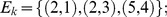

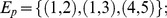

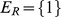
 as only the layer 1 has intra-layer links.

The alternative representation 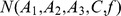 of the SBN example is given as follows.

- The adjacency matrix *A*_1_ for the “TTC” layer (layer 1) is defined as:

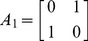
(8)
- The adjacency matrices *A*_2_ for the “supporting subsystems” layer (layer 2) and *A*_3_ for the “payload” layer (layer 3) are trivial as there is no intra-layer links in these layers:

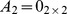
            and 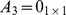
(9)

- The inter-layer matrix *C* is as follows:
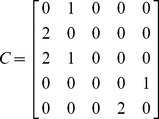
(10)

The overall numbering scheme can be chosen to facilitate the representation of the IMLN, and in particular the inter-layer matrix *C*. If the “overall numbering” is chosen such that nodes belonging to the same spacecraft are numbered sequentially (nodes 1, 2 and 3 belong to S/C#1, and nodes 4 and 5 to S/C#2) as in the present case study, the inter-layer matrix *C* can be reduced to a block diagonal form:
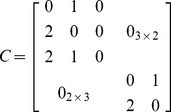
(11)


As the number of spacecraft increases in the space-based network, the inter-layer matrix growth can be alleviated using this numbering scheme, as only the blocks around the diagonal need to be populated. Also, from a computational point of view, this can allow for the matrix to be saved as a scarce matrix and save memory during the simulation.

Finally, the mapping function *f* for the SBN example is given by:

- In the “TTC” layer, numbered layer 1, the node 1 in the “overall numbering” is given the “layer number” 1, while the node 4 in the “overall numbering” is given the “layer number” 2;
- In the “supporting subsystems” layer, numbered layer 2, the node 2 in the “overall numbering” is given the “layer number” 1, while the node 5 in the “overall numbering” is given the “layer number” 2;
- In the “payload” layer, numbered layer 3, the node 3 in the “overall numbering” is given the “layer number” 1. Then the mapping function *f* is:

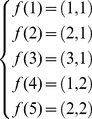
(12)This latter representation is easily scalable for larger networks, as shown in Castet [22] for a network with four layers and ten super-nodes: the adjacency matrices and the inter-layer matrix are scarce, and consequently easily handled. A complete study of the scalability of the interdependent multi-layer network approach is provided in Castet [22]. The next section examines failure propagation across interdependent multi-layer networks or equivalently across networks with heterogeneous nodes.

### Failure Propagation in Interdependent Multi-Layer Networks

Assessing the survivability of an interdependent multi-layer network requires estimating an objective function related to the failure times of the nodes in the network. Due to the interdependencies in the model, this estimation is not trivial and requires careful accounting of various issues discussed next. Part of the failure propagation is due to both the kill effect and the precursor effect. The following subsections examine in details these effects. The proposed method comprises four steps:

1. Generate the times to failures *T_F_* for each node and intra-layer link;
2. Propagate failures through inter-layer links related to the kill effect;
3. Propagate failures through inter-layer links related to the precursor effect;
4. Combine all effects to obtain the probability of unavailability of each node.

A mathematical characterization of these steps is provided next. The interdependent multi-layer network of interest is defined as 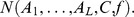 In this work, node and link complete failures are investigated. The treatment of partial failures and anomalies can be found in Castet [22].

### 3.1. Time to Failure Generation

To propagate failures through the network, one must first generate times to failures for different objects in the space-based network: the nodes and the intra-layer links. Using the cumulative distribution functions representing the failure behavior of each node, random times to failure for the nodes *T_F,_*_node *i*_ 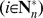 can be generated. Note that it is not necessary for each node in a common layer to share the same failure behavior (diversity of redundancy).

Two steps are needed to generate the times to failure for the intra-layer links *T_F,_*_link *j→i*_: the link between two spacecraft is established through a wireless unit embedded in each spacecraft. For the link to function, both units need to be operational, the failure of one leading to the failure of the link.

- Generate the times to failure of the wireless units on each spacecraft using predetermined cumulative distribution functions;
- Generate the times to failures for each intra-layer link *T_F,_*_link *j→i*_ by taking the minimum of the time to failures of the two associated wireless units (unit *i* and unit *j*).

### 3.2. Failure Propagation through the “Kill Effect”

The information about the kill effect is contained in the inter-layer matrix *C*, and the first step consists in extracting from *C* the pairs of “killer” and “victim” nodes. As shown previously, *E_k_* can be defined from *C* as follows:
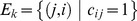
(13)


Define the “killer” vector **k_1_** and the “victim” vector **v_1_** such that:
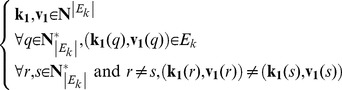
(14)


The last step consists in computing time to unavailability 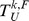 of the “victim” node using the time to failure of the “killer” node. Mathematically, this is expressed as:



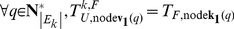
(15)


In the case that a victim node has several killers, 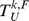 is the minimum of the times to failure of the killer nodes.

### 3.3.Failure Propagation through the “Precursor Effect”

In a similar vein to the killer effect, the information about the precursor effect is contained in the inter-layer matrix *C*, which is used to extract the pairs of “killer” and “victim” nodes. We define *E_p_* as follows:
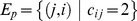
(16)


The “killer” vector **k_2_** and the “victim” vector **v_2_** are defined as:
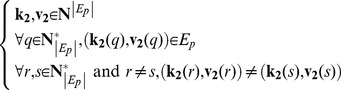
(17)


Computing the time to unavailability due to the precursor effect is not as straightforward as for the kill effect. Indeed, the failure of a node that has a functional redundancy will not necessarily propagate immediately to the nodes belonging to the same entity (here, spacecraft). The time at which the function represented by the node will become unavailable depends on the time to failure of the node itself, and on the times to failure of the other nodes and links in the same layer. For example, for the SBN presented in Figure 4, the failure of node 1 will propagate to nodes 2 and 3 if node 1 is not able to tap into the resources of node 4, that is, if either the link between node 4 and 1, or node 4 failed. Hence it is necessary to compare the time to failure of the node to the ones of the pairs link/node it is connected to. Several steps are needed and they are described next:

1. To know when a node becomes unavailable after the kill effect, the “minimum time to unavailability” 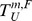 is introduced and is defined as:

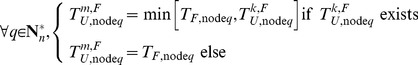
(18)
2. To compare the time to failure of the node *i* and the ones of the pairs link (*j*→*i*)/node *j* it is connected to (links *towards* that node *i*), a convenient mathematical object is introduced, the matrix 
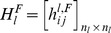
 defined as follows for 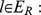

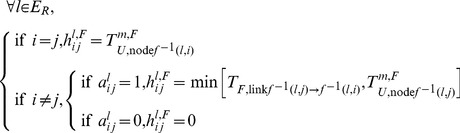
(19)
  This matrix is helpful as it presents in the same row the time to failure of the node, and the ones of the pairs link/node it is connected to.
3. The time to unavailability considering the functional redundancy 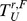 of the node of interest can be found as the maximum time to failure in the associated row. Consider the column vector 
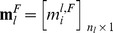
 defined for 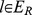 as:
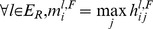
(20)

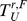
 can now be computed as:

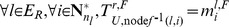
(21)
5. The same process as that of the previous kill effect can be now applied to examine the propagation of the “failure” of a node across layers to nodes belonging to the same super-node. This step consists in computing time to unavailability 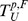 of the “victim” node using the time to failure of the “killer” node. Mathematically, this is expressed as:

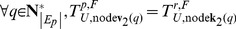
(22)
  In the case that a victim node has several killers, 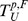 is equal to the minimum of the times 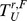 of the killer nodes.
6. Due to the fact that several layers of redundancy can be considered concurrently, the interdependence of the precursor effect between nodes belonging to the same super-node but in different layers can require an iterative scheme for unavailability times to converge to their correct values. The following condition indicates if more iterations are required: if 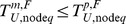 the failure propagation due to the precursor effect is complete (skip step 7 and continue to subsection 3.4). If not, continue to next step.
7. While 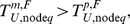 set 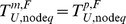 and repeat steps 2–5.

### 3.4. Combination of Kill and Precursor Effects

Finally, for each node in the interdependent multi-layer network, the time to unavailability is obtained by combining the information about the time to failure of the node itself, the potential additional survival time enabled by the node connectivity (Eq. (21)), and the unavailability times imposed on the node from the kill and precursor effects (Eqs. (15) and (22)) as:



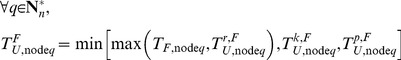
(23)where 
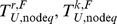
 and 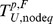 are included in if they exist.

### 3.5. Summary of the Failure Propagation Algorithm

A summary of the algorithmic process used to propagate failures across the network is here provided. The following inputs are required: the adjacency matrices and inter-layer matrix, the mapping function (these three elements defining a network architecture), and the CDFs for the failure distribution of the nodes and links.

1. Generate for each node *i T_F,_*_node *i*_
2. Generate for each link *T_F,_*_link *j→i*_
3. Compute *E_k_* using Eq. (13)
4. Compute **k_1_** and **v_1_** using Eq. (14)
5. Compute 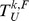 for each victim node using Eq. (15)
6. Compute *E_p_* using Eq. (16)
7. Compute **k_2_** and **v_2_** using Eq. (17)
8. Compute 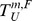 for each node using Eq. (18)
9. For all 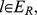 compute 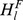 using Eq. (19)
10. For all 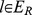, compute 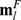
using Eq. (20)
11. Compute 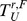 for each node for all layers 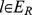 using Eq. (21)
12. Compute 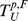 for each victim node using Eq. (22)
13. Repeat steps 9–12 until 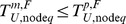 for all victim nodes *q* in the precursor effect
14. Compute 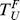 for each node using Eq. (23)

This approach has been carefully validated, and the reader is referred to Castet [22] for detailed analyses and validation.

## Applications and Illustrative Results

### 4.1. TTC Functional Redundancy Case with Perfect Wireless Link

The first space-based network considered is the simple example used previously (Figure 4), which consists of a network of two spacecraft that can share their TTC resources. This particular example was selected as previous studies have identified the TTC subsystem as a major driver of spacecraft unreliability [23], therefore spacecraft in a network would likely benefit for being able to tap into each other's TTC. The focus in this illustrative case is on endogenous failures, and as a consequence, the survivability results are limited to this particular class of threat, and they should not be extrapolated to other classes of on-orbit shocks. The failure behaviors of the different spacecraft subsystems are summarized in Table 1 (see Castet [22] for the derivations).

**Table 1:** Weibull parameters for spacecraft subsystems' failure times in the TTC case.

Functionality	Weibull shape parameter *β*	Weibull scale parameter *θ*
		year
Telemetry, Tracking, and Command (TTC)	0.4650	47,770
Supporting subsystems	0.5529	918.5
Payload	0.5921	30,150

The survivability analysis of the interdependent multi-layer network shown in Figure 4 examines the utility generation capability of the space system, that is, the probability that the payload node (node 3) remains fully operational, or alternatively, the probability that it becomes unavailable. As a consequence, the metric of interest is 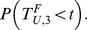 The survivability results here presented are limited to this metric, but they can be easily expanded to other performance metrics of interest.

**Figure 5:**
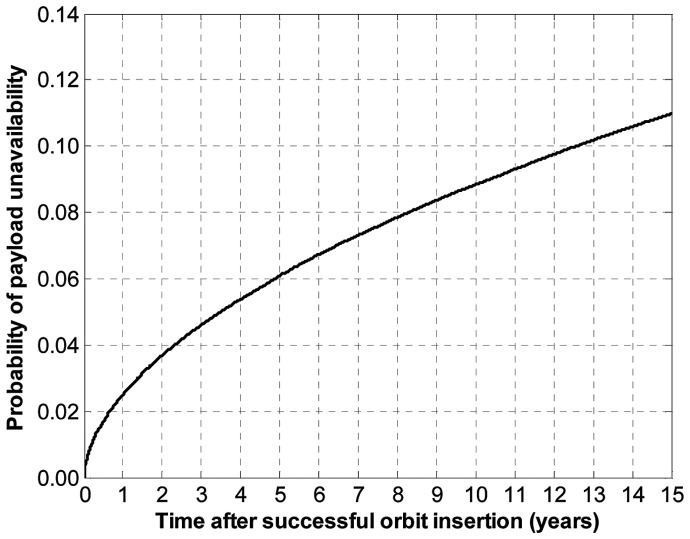
Output probability of payload unavailability with TTC functional redundancy.

Assuming first a perfectly reliable wireless link between spacecraft and running a Monte Carlo simulation yield the probability of unavailability of the payload node shown in Figure 5. Figure 5 reads as follows: for example, after 5 years on orbit, there is about 6% chance that the payload will be unavailable to generate utility. This probability increases to about 9% after 10 years on orbit.

**Figure 6:**
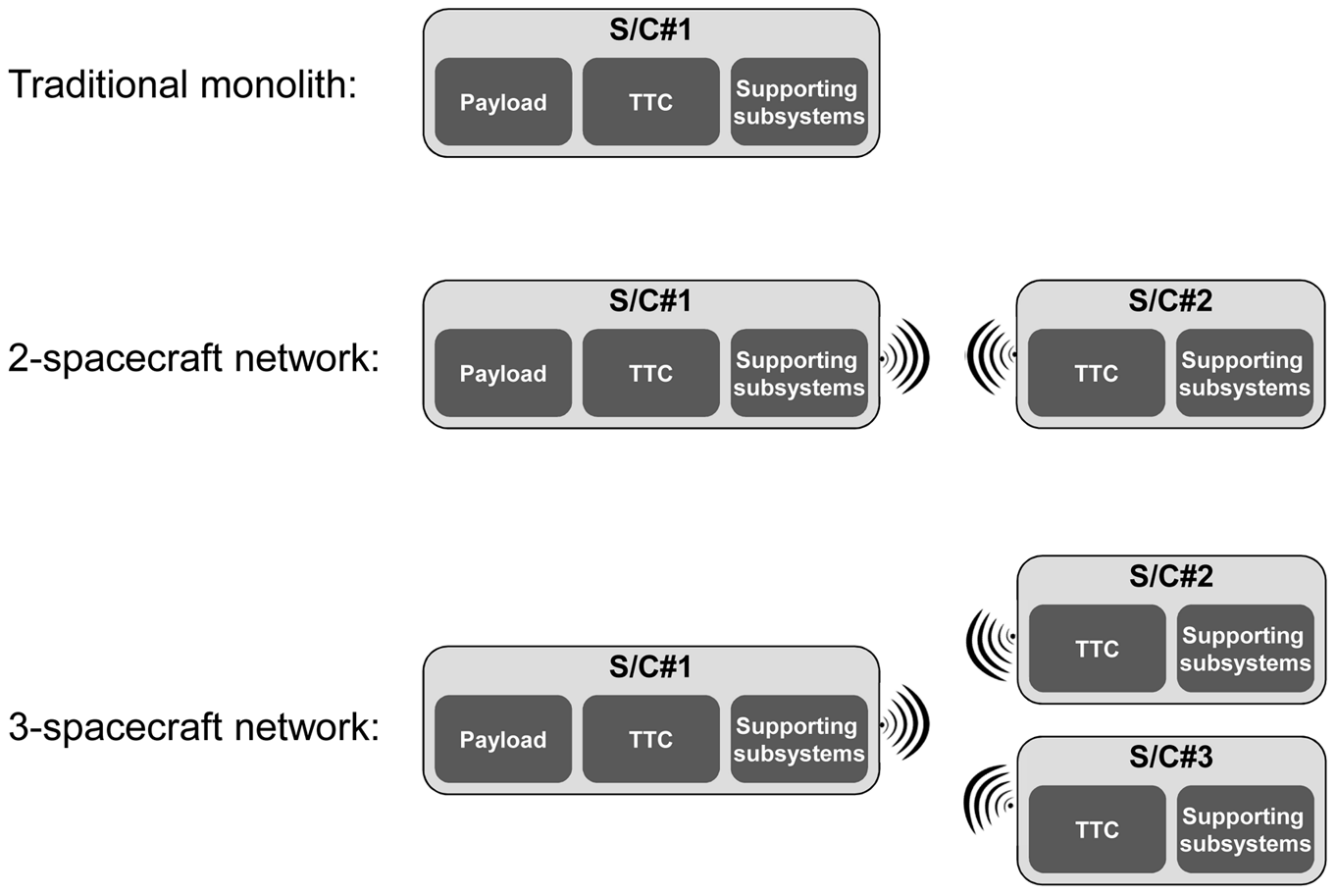
Space architectures with different levels of TTC redundancy.

**Figure 7:**
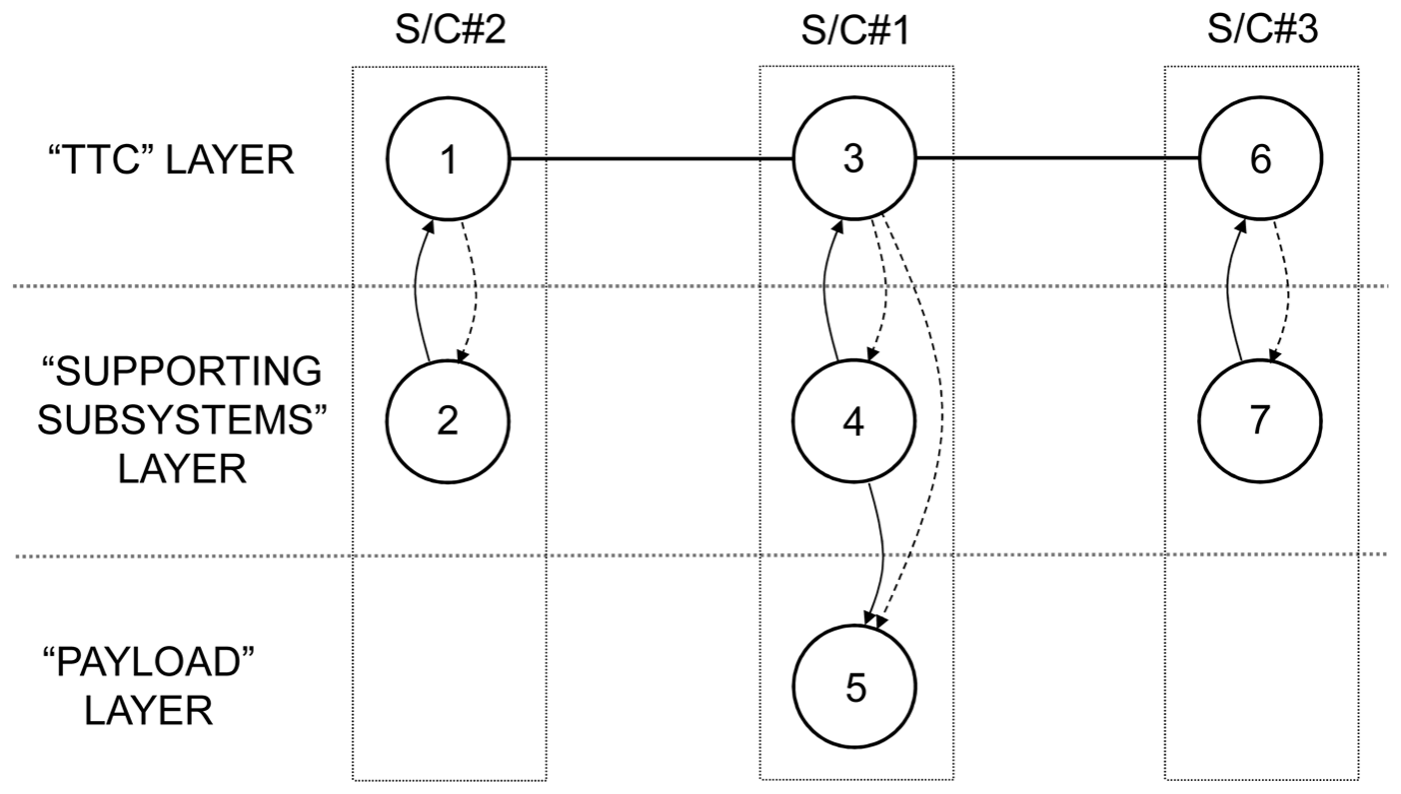
IMLN representation of the space-based network with 3 spacecraft for TTC functional redundancy.

**Figure 8:**
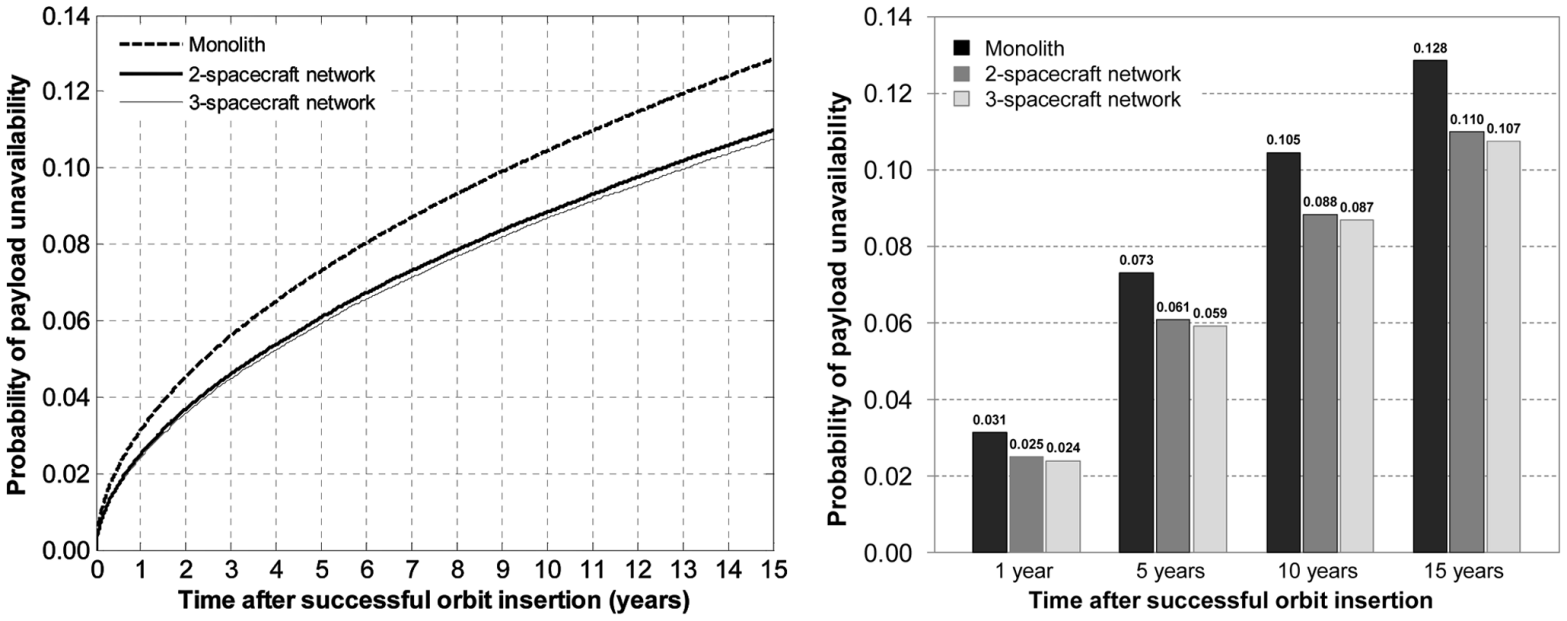
Comparison of the probabilities of unavailability of the payload for a single, two-spacecraft and three-spacecraft architectures.

In addition to such results as shown in Figure 5, the method here proposed allows to quickly conduct a comparative survivability analysis of different architectures. For example, two additional architectures are examined next: the traditional monolith spacecraft and a three-networked spacecraft architectures, presented in Figure 6 alongside the two-networked spacecraft studied above. The interdependent multi-layer network representation for the three-spacecraft network is shown in Figure 7 and the associated matrices and mapping function are the following:

– Adjacency matrices: 
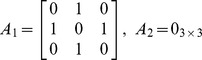
and 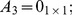


– Inter-layer matrix: 
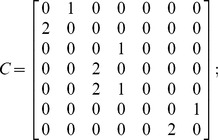


– (Inverse) mapping function: 
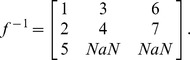


Assuming perfectly reliable wireless links between spacecraft, the probabilities of payload unavailability for each of the three architectures are shown in Figure 8. Figure 8 reads as follows: after 15 years, there is a 12.8% chance that the traditional monolith spacecraft will unavailable (no utility generation), compared with an 11.0% for the two-spacecraft network, and a 10.7% for the three-spacecraft network. In other words, adding spacecraft to the network, with TTC redundancy, will increase the survivability aspect of the architecture with respect to endogenous failures. The modeling approach and analysis here proposed quantify the extent of survivability of different network architectures. In this example, the decrease in the probability of total failure of the payload node (or node unavailability) for the two-spacecraft network architectures represents a 14% variation compared with the probability of total failure of the monolith architecture, which can be considered a significant improvement over the current spacecraft design paradigm. A significant share of the difference occurs early in the life of the space-based network, consistent with the fact that most spacecraft subsystems suffer from infant mortality [24].

A careful cost-benefit analysis should be conducted to assess whether this incremental probability of generating utility is worth the cost of obtaining it by designing, manufacturing and launching additional spacecraft. While such studies are beyond the scope of this work, it is worth pointing out that communication satellites for example can generate in excess of $50 million per year and these increments in lowering the probability of failure can represent the equivalent of several months' worth of revenues. Similarly, this survivability increment can be of significant importance for defense and intelligence space assets.

Figure 8 shows a minor incremental benefit of 3-spacecraft over the 2-spacecraft network under the assumptions here considered (endogenous failures and perfect wireless link): adding one spacecraft to the traditional monolith spacecraft for TTC functional redundancy decreases the probability of payload unavailability by 1.8 percentage points, but adding two spacecraft to the monolith for the same purpose decreases this same probability by 2.1 percentage points. The ability to generate such findings is important for system engineers in assessing the cost and quantifying the benefits of different network design alternatives.

### 4.2. Impact of the Wireless Link Failure

The assumption made previously of a perfectly reliable wireless link between spacecraft may not be justified in practice, and, as a result, the survivability advantages of the space-based network over the monolith spacecraft may not be fully realizable. To assess the impact of the wireless link failure, a wear-out failure behavior for the link is injected in the simulation. The probability of failure of the link is labeled *υ_F_*(*t*). Assuming that both wireless units for the network in Figure 4 are identical and that their failure is modeled using a Weibull distribution with a shape parameter *β* and a scale parameter *θ*, the probability of failure of the link is given by:
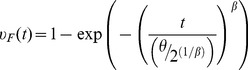
(24)


**Figure 9:**
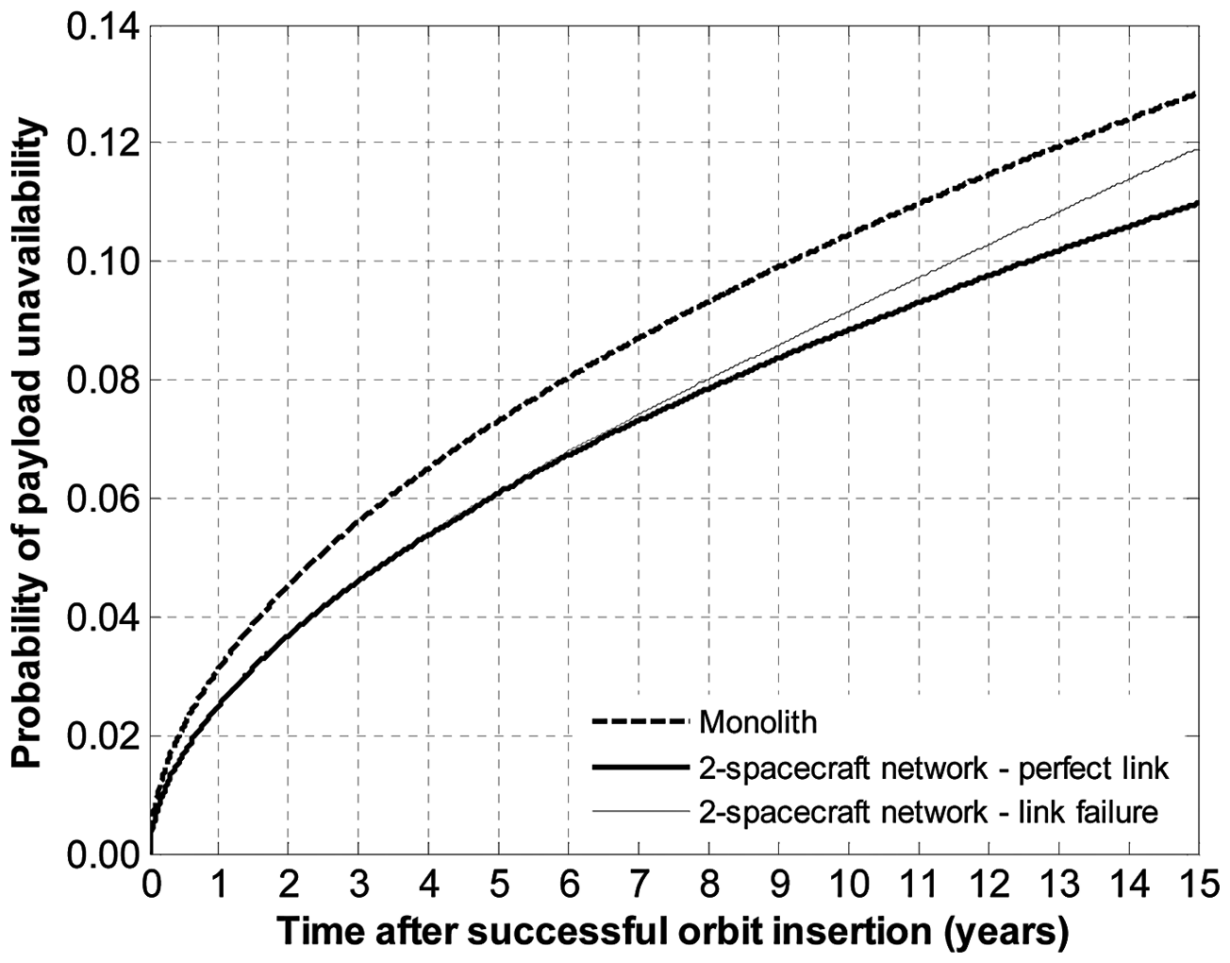
Impact of an imperfect wireless link.

We examined a wide range of parameters for the Weibull failure behavior of the wireless link, exhibiting both infant mortality and wear-out failures. The results shown in Figure 9 are for the representative Weibull parameters *β* = 3 and *θ* = 21.36 years that result in a 50% chance of link failure after 15 years on-orbit. Figure 9 shows the probability of unavailability of the payload node alongside the results for the monolith architecture and the two-spacecraft network architecture with a perfect link. As such, Figure 9 clearly identifies the effect of the wireless link failure.

Figure 9 show how the probability of payload unavailability is impacted by the unreliability of the link. In the case shown here, with the link exhibiting wear-out failures, we note, as expected, that the probability of failure for the two-spacecraft network architecture with an imperfect link diverges from its ideal case late in the system's life on orbit (around year 7 in the figure). The more general finding is that the survivability advantage of the space-based network accrues before the link begins exhibiting failures, and that the more likely the link will fail, the smaller the survivability advantage (more divergence from the ideal case). Infant mortality of the wireless link (prevalence of early failures) blunted the survivability advantage of a space-based network early on, and as a result, rooting out such failure behavior of the wireless link, through burn-in or other procedures, ought to be a high priority for designers and manufacturers if space-based network are to offer a sustained advantage over the traditional monolith spacecraft design.

### 4.3. C&DH Functional Redundancy Case with Perfect Wireless Link

The previous example with the TTC redundancy showed a simple example to demonstrate the capability and the use of the approach here introduced. This approach can handle more complex architectures that are beyond the capability of current reliability tools. As an example, a more complex architecture is presented next. Other spacecraft subsystems can be selected for sharing on-orbit resources: for example, the Control Processor (main computer of the spacecraft) is a good candidate as spacecraft could pool their processing power, or one spacecraft could run processes and command another spacecraft if the Control Processor (CP) subsystem of that spacecraft failed, if sufficient processing power margin is built into the supporting spacecraft. An additional fractionable subsystem is the Data Handling subsystem (DH) (responsible for storing and exchanging data): for example, one spacecraft can be envisioned as the “hard drive” of the constellation, on which networked modules upload their data, data then downlink to the ground station by the collector spacecraft. The macro subsystem combining the TTC, the CP and DH is also referred to as the Command and Data Handling (C&DH) subsystem.

**Figure 10:**
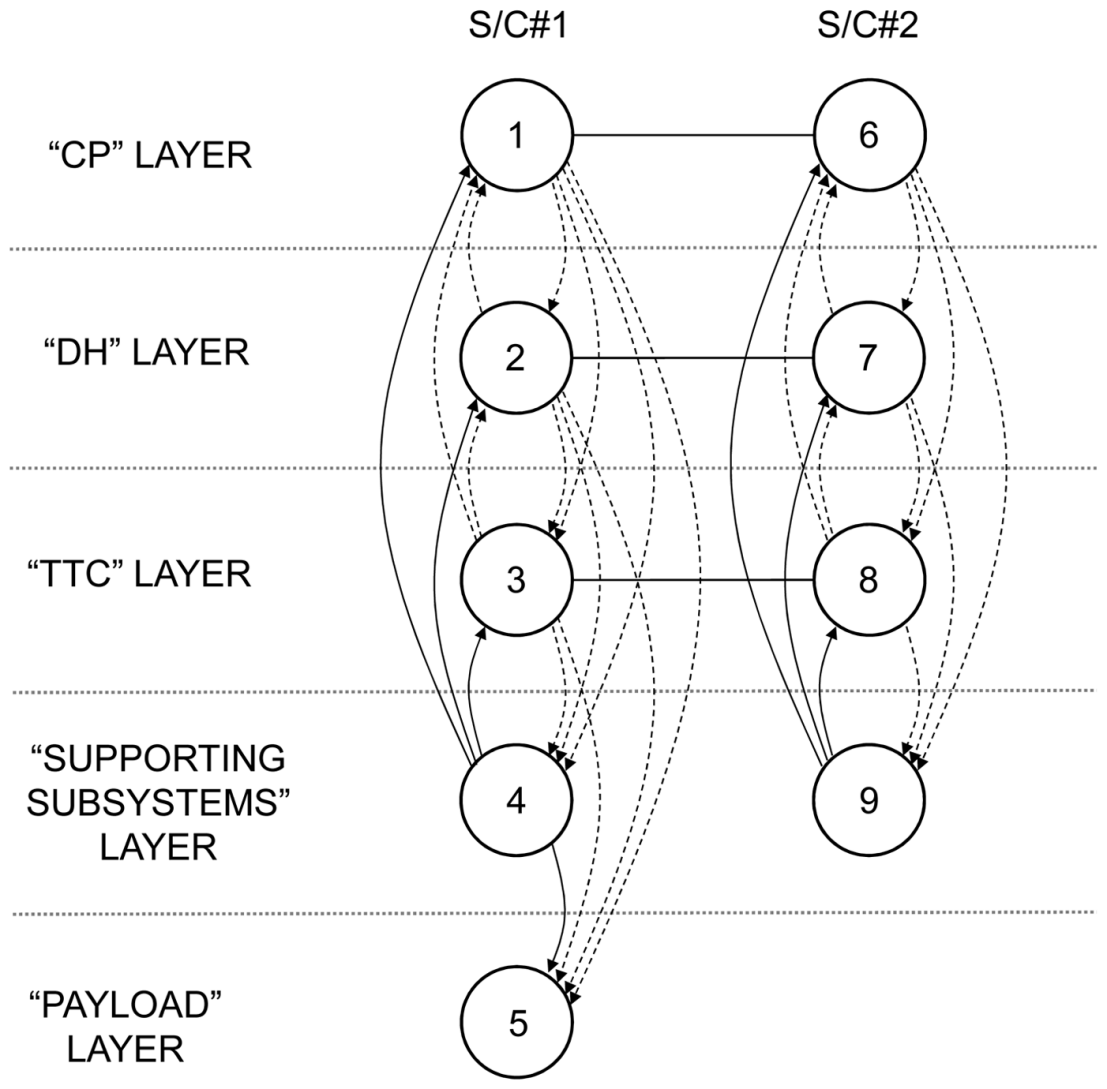
IMLN representation of the space-based network with C&DH redundancy.

The interdependent multi-layer model needs to account for these new separate functionalities: there are now five functionalities: the CP, DH, TTC, supporting subsystems, and payload. As a consequence, the network representation will consist of five layers, one for each of the aforementioned functionalities. Two spacecraft are part of the network: the first spacecraft has all the subsystems, while the second has all the subsystems except the payload and acts as a functional redundancy for the first spacecraft for the CP, DHS and TTC. The associated representation is shown in Figure 10, and the adjacency and inter-layer matrices as well as the mapping function are provided next. The failure behaviors of these functions (nodes) are summarized in Table 2 (see Castet [22] for the detailed derivations).

**Table 2:** Weibull parameters for spacecraft subsystems' failure times in the C&DH case.

Functionality	Weibull shape parameter *β*	Weibull scale parameter *θ*
		year
Control Processor (CP)	1.251	691.2
Data Handling (DH)	0.6266	350,000
Telemetry, Tracking, and Command (TTC)	0.4650	47,770
Supporting subsystems	0.5529	918.5
Payload	0.5767	49,990

**Figure 11:**
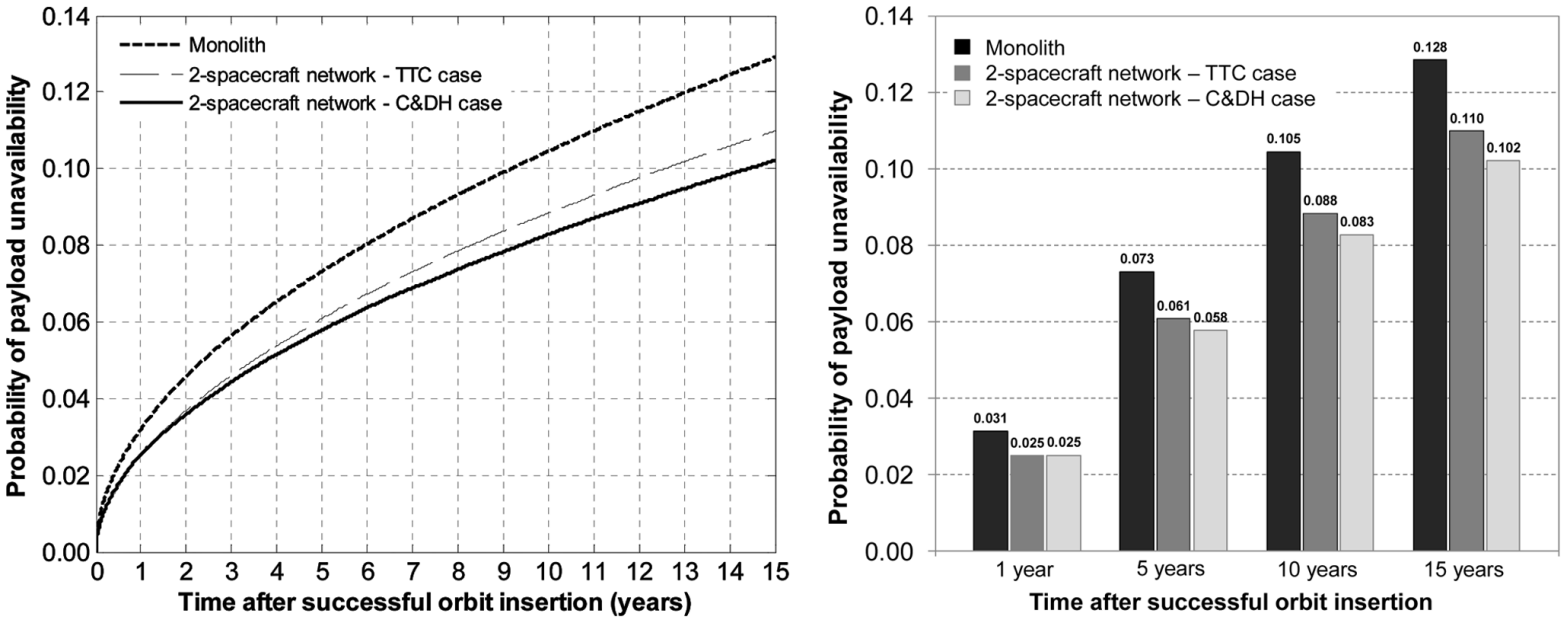
Output probability of payload unavailability with C&DH functional redundancy alongside with the monolith case and the TTC case.

– Adjacency matrices: 
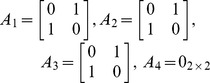
 and 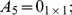


– Inter-layer matrix: 
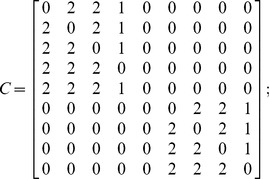


– (Inverse) mapping function: 
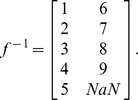


Assuming a perfectly reliable wireless link between spacecraft and running a Monte Carlo simulation yields the following results presented in Figure 11, superimposed on the cases shown previously: the traditional monolith architecture and the TTC functional redundancy case.

Figure 11 demonstrates the survivability improvements brought by networking spacecraft on-orbit and providing them with the ability to tap into each other's C&DH subsystems. For example, it can be seen that after 15 years, the network decreases the risk of payload unavailability by 2.6 percentage points compared with the monolith spacecraft. This represents a 20.5% decrease in the risk of losing payload utility (with respect to C&DH endogenous failures), which is one additional argument, on the benefit side, in favor the SBN. As noted previously, all the benefits, survivability and others, provided by networking spacecraft on-orbit have to be carefully weighted against the costs and risk of doing so. Preliminary comparative analyses of cost and utility of SBN and monolith spacecraft can be found in Dubos and Saleh [19].

The current state-of-the art in space technology readily supports the fractionation and networking of the C&DH subsystem and its constitutive elements. Other subsystems such as the Electrical Power (EPS) and the Attitude and Orbit Control (AOCS) subsystems would require technological breakthroughs before they can be networked.

## Conclusion

This work introduced a novel approach and algorithmic tools for the modeling and survivability analysis of networks with heterogeneous nodes. The research was motivated on the one hand by the perceived limitations of the traditional network survivability analyses, which assume for the most part node homogeneity (or some variations on the same function), and on the other hand a growing interest in space-based networks in which different nodes (spacecraft) can share various on-orbit resources with neighboring spacecraft.

The proposed approach is based on the idea of mapping a network with heterogeneous nodes into an interdependent multi-layer network (IMLN). The multi-layer aspect captures the breakdown of the network according to common functionalities across the different nodes and allows the emergence of homogeneous sub-networks, while the interdependency aspect constrains the network to capture the physical characteristics of each node. Formal definitions of the IMLN representation as well as primitives of failure propagation across the network were developed in support of the survivability analysis of the network under consideration. An algorithm for the propagation of node and link failures was also provided.

This approach and the tools developed were applied to the case of space-based networks (SBNs), which consist of several networked spacecraft that can share on-orbit resources. The case studies examined included networked spacecraft that can share their Telemetry, Tracking and Command (TTC), their Control Processor (CP), and their Data Handling (DH) sub-subsystems. Results quantified the survivability gains exhibited by the SBN over the traditional monolith spacecraft, and they highlighted among other things the importance of the reliability of the wireless links between spacecraft (nodes) to achieve these gains.

The application here considered was confined to spacecraft endogenous failures, but it can be adapted to other classes of threats (orbital debris or ASAT weapons) once their probabilities of occurrences are modeled.

Analysts and system designers may be interested in assessing the impact of varying the failure behavior of a non-descriptive node/functionality on the survivability performance of different network architectures. The tools here presented enable an easy analysis of such consideration by parameterizing the failure behavior of one or more nodes, and assessing the resulting survivability performance of various networks for wide range of parameters. As a result, different network architectures can be ranked with respect to their survivability performance (in response to specific classes of threats), which in turn can help inform decision-making with respect to the selection of particular network features, e.g., how many nodes, what type of meshed/star/other network architectures are more appropriate from a survivability perspective for a given class of threats. These issues are explored in forthcoming publications and they continue to offer many fruitful venues for further research.

Finally, it is worth noting what goes without saying that the survivability advantages obtained though networking spacecraft on-orbit should be carefully assessed and traded against the incremental costs and risks of achieving them.
